# Selected Research Issues of Urban Public Health

**DOI:** 10.3390/ijerph19095553

**Published:** 2022-05-03

**Authors:** Judith Schröder, Susanne Moebus, Julita Skodra

**Affiliations:** Institute for Urban Public Health, University Hospital Essen, University Duisburg-Essen, 45130 Essen, Germany; susanne.moebus@uk-essen.de (S.M.); julita.skodra@uk-essen.de (J.S.)

**Keywords:** urban public health, urban environments, health resources

## Abstract

Health is created within the urban settings of people’s everyday lives. In this paper we define Urban Public Health and compile existing evidence regarding the spatial component of health and disease in urban environments. Although there is already a substantial body of single evidence on the links between urban environments and human health, focus is mostly on individual health behaviors. We look at Urban Public Health through a structural lens that addresses health conditions beyond individual health behaviors and identify not only health risks but also health resources associated with urban structures. Based on existing conceptual frameworks, we structured evidence in the following categories: (i) build and natural environment, (ii) social environment, (iii) governance and urban development. We focused our search to review articles and reviews of reviews for each of the keywords via database PubMed, Cochrane, and Google Scholar in order to cover the range of issues in urban environments. Our results show that linking findings from different disciplines and developing spatial thinking can overcome existing single evidence and make other correlations visible. Further research should use interdisciplinary approaches and focus on health resources and the transformation of urban structures rather than merely on health risks and behavior.

## 1. Introduction

Urban Public Health gains in importance due to increasing health challenges of the ever-growing urban population. Many authors agree that we have entered the ‘urban age’ [1,2,3,4] despite the lack of consensus over the definition of urban areas [2]. Urban areas are the places and arenas of important societal changes and struggles around healthy environment, climate change, social justice, or the future of work and mobility. Urbanisation is coupled with different challenges related to Urban Public Health on the Global North (noncommunicable diseases and urban regeneration) and on the Global South (communicable diseases, urban expansion, and unfavourable living conditions) [4]. Multiple interdependencies on the global level influence cities and neighbourhoods that are local and context depended, but also local actions and activities may cause global crisis. Recognising the important role of urban setting, in the last decades, there have been calls to transform our cities into sustainable, healthy, and just places for global population [5,6]. Meanwhile, good health and wellbeing is one of the sustainable development goals (SDG3) [6], a cross-cutting issue of all other SDGs [7], of the New Urban Agenda [8], and a core issue in a recent publication from the UN-Habitat and World Health Organisation (WHO) on good planning of urban environments [9].

In view of global urbanization trends, the question how to shape urban environments should be closely linked to the health of the urban population [9]. Urban planning and governance have great impact on the distribution of health-promoting resources and on accumulation of risks that affect health of different population groups [10,11,12,13,14,15,16,17] and contribute to both communicable and non-communicable diseases [18]. The importance of urban environments for health has been known since the ancient Greek city-states around 500 BC [19,20]. However, during the 20th century, health and disease were explored and practiced in a predominantly biomedical and individual-oriented approach. Lastly, with the Ottawa Charter (1986), a paradigm shift was initiated from an individual-oriented to a structural perspective emphasizing the importance of everyday settings in terms of health promotion [21]. Moreover, the Ottawa Charter shifted the focus from the causes of disease to a new understanding of health as resource and health promoting factor. The Charter emphasized for the first time that the responsibility for health also lies within many sectors besides health.

Meanwhile it is recognized that the urban living environment is not a self-contained, homogenous entity, but a complex system characterized by a number of different urban structures (e.g., educational, economic, mobility, political structures) that have their own dynamics and interact with each other in a complex urban grid. Thus, improving health and preventing disease in urban environments require genuinely joint efforts of different disciplines and sectors [22]. As an interdisciplinary research field, Urban Public Health attempts to address this need. The main aim of Urban Public Health is to explore the dimensions of health and disease in and by urban structures [15] (pp. 342–343). The international scientific debate, which operates primarily with the term Urban Health has its theoretical and disciplinary roots mainly in human ecology or medicine [10,23,24]. In contrast, we understand Urban Public Health explicitly as part of public health. This allows us to build on existing and well-established constructs and methods of public health, which include epidemiological tools. We extend the public health approach by linking it to the specific features of urban structures, paying particular attention to spatial relationships.

Addressing the spatial component allows insights into the distribution and constitution of health resources and risk factors in or by urban structures. The underlying question is how cities can be designed and (re)developed to create urban structures as health resources. Although there is already a substantial body of single evidence on the links between urban environments and human health, less is known regarding the specific connection between public health and place-based associations. We want to address this gap and explore in this paper the extent to which evidence on health effects of different urban structures contribute to Urban Public Health field by linking spatial approaches and public health.

Moreover, a conceptual overview of this fairly new field of Urban Public Health and its open research questions is still missing. Accordingly, the aim of this paper is to review current evidence regarding the spatial component of health and disease in urban structures and to identify research issues, with particular attention to the links between public health and spatial perspectives from other disciplines such as urban planning, geography, social, and political science. In particular, our approach is not limited to looking at health outcomes and exposures, but rather we ask through a structural lens about the living conditions in urban environments beyond individual health behaviors. It is not within the scope of this article to comprehensively review findings for the entire field of Urban Public Health, but rather to present selected evidence from the view of the professional backgrounds of the authors, which is political science, urban planning, public health, and epidemiology. The aim is to provide a narrative towards new insights and possible research issues that can contribute to better understanding of the Urban Public Health challenges and potentials.

### Categorization of Urban Public Health Issues

The complexity of interactions between urban environments and human health [25] is already conceptualized in several frameworks. Well-known ones are the *Conceptual Framework for Urban Health* [14] from Galea et al., the *Health Map* of Barton and Grant [11], and the illustration of *Health Problems in different urban contexts* from Rydin et al. [17]. A more recent example is the *Conceptual Model of Key Drivers of Urban Health, Equity, and Sustainability* [10]. They all describe, inter alia, how a variety of urban structures influences and shapes health and disease.

Based on these frameworks, we organized our review by selecting and categorizing Urban Public Health issues. We used three main categories: (i) *build and natural environment*, (ii) *social environment*, and (iii) *governance and urban development*. The first two categories include subcategories as listed in Table 1. Since the transformation of urban environments into sustainable places and the shaping of health resources is to a considerable extent a matter of political negotiation processes, we defined the third category *governance and urban development.* This category goes across the first two categories. We compiled knowledge describing political structures that are shaping and influencing the built, natural, and social environments.

The built and natural environment describes the physical-material level of urban structures as material expressions of human activity and societal constitution. Research here is concerned with relations between physical space and health. The social environment describes the characteristics and properties of communities in and by urban structures, the prevailing social norms, processes of exchange and interaction, and their relation to health. Aspects of the social and physical environment interact with each other and are interdependent [15]. How these environments interact with each other and how they are shaped largely takes place on the basis of prevailing governance structures.

This paper is divided into five sections. The next section introduces the methods we used to create a compilation, select papers, and analyze results. We then critically review and describe the selected categories of Urban Public Health. This is followed by the discussion and more detailed examination of existing gaps. We conclude with the most critical aspects and recommendations for future research.

## 2. Materials and Methods

**Figure 1:**
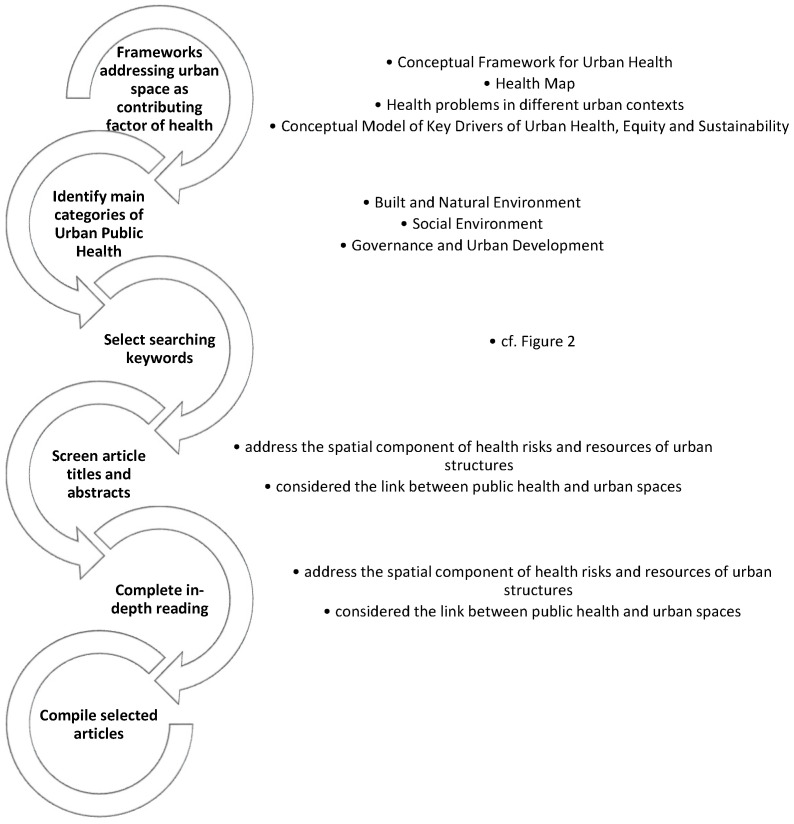
Methodical approach and search strategy for the narrative review.

**Figure 2:**
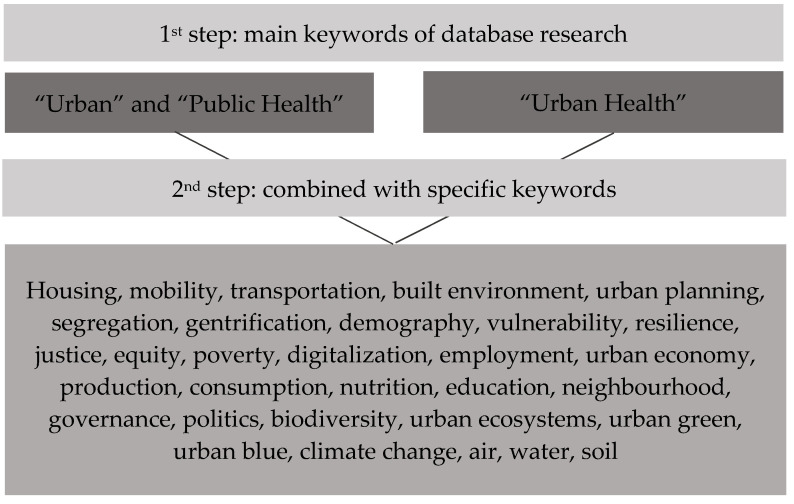
Keywords of database research and research strategy.

In order to narrow down the project we proceeded in three steps. In step 1, we outlined the conceptual approach for our review by using frameworks addressing urban space as a contributing factor for health [10,11,12,13,14,15,16,17]. In step 2, we categorized the main topics of Urban Public Health, and used them to assign and compile the articles in step 3. Between February and May 2021 we screened the literature by performing a snowball approach in the databases PubMed, Cochrane, and Google Scholar (a tabular overview of the main literature for analysis can be found in the Appendix A, Table A1). As a restriction, the language filter was set to English. The methodical approach and search strategy of the narrative review is also illustrated in Figure 1. Instead of reviewing singular results of studies on single public health aspects of urban environments, we focused our search to existing review articles and reviews of reviews for each of the used keywords in order to cover the range of issues in urban environments. The study area is focused on developed countries. We used the main keywords “Urban”, “Public Health”, and “Urban Health”. The search was refined by varying terms and combinations using predefined specific keywords (Figure 2).

The results were filtered by the first author by screening article titles and abstracts to determine: (a) if they address the spatial component of health risks and resources of urban structures, and (b) if they considered the link between public health and urban spaces. As a result of this screening, 183 papers were selected for a complete in-depth reading. According to the abovementioned criteria, (a) and (b), the final articles were selected for analysis. Finally, we included 120 papers for our review. As our approach is not a systematic review, we refrained from independent assessment of identified reviews. This approach has some limitations: important scientific contributions may be missed due to the selection criteria, e.g., (i) the selection of key words; (ii) the missing double check of excluded articles; (iii) by opting for English-language articles only. However, overall, this approach allowed us to explicitly address prevailing research strands and broad lines of Urban Public Health in order to identify existing research gaps at a more fundamental level.

## 3. Results

### 3.1. Built and Natural Environment

#### 3.1.1. Overarching Issues

The built form of the city is a visible expression of the complex urban structures and socio-cultural characteristics of the heterogeneous population. Evidence shows that urban environment has direct and indirect impacts on physical and mental health [26,27]. However, urban environment is also conditioned by understandings of health in the sense of socio-cultural and historical specifications of health, which have constitutive effects on the built form [20,23]. Main characteristics of the built environment that are related to the population health are: buildings and density, land use, scale of streets and streets network, local facilities (services and retail), and public open spaces [11,14]. Mix of land uses, as well as design and maintenance of the urban environment, may support a healthy lifestyle and contribute to improved physical and mental health [28]. Design strategies determine the connection between morphological and functional features of urban environment that may provide opportunities for public health promotion and protection [29], but if inadequate, may also impair health. It has been well known that aspects such as high density, inadequate housing, and poor water supply and sanitation promote vector proliferation [30]. In the last decades, non-communicable diseases, such as cardiovascular diseases, diabetes, and mental health problems, are taking over the vector diseases [31,32,33] and can be associated with the build environment [34,35].

Although there is a general trend of increasing life expectancy [31,36] (p. 29), substantial inequalities in life expectancy between deprived and privileged neighborhoods still remain a challenge [37]. In particular, socioeconomic inequalities in relation to different components of the built environment have come to the fore. For example, Gelormino et al. [38] highlight key features of the built environment that shape the health of its inhabitants. However, these key features are unequally distributed and closely related to socioeconomic status [38]. Moreover, Dendup et al. [39] show in their review associations between development of Type 2 Diabetes mellitus and a health promoting urban environment, such as walkability, air quality, opportunities to easily purchase healthy foods and a range of facilities for physical activity. However, they acknowledge that there is still a lack of evidence on the influence of socioeconomic or demographic factors on the relationship between the environment and type 2 diabetes. Beyond diabetes, mixed land use, pedestrian and bicycle-friendly infrastructure, and street connectivity, as well as green and open spaces also have positive connotations with physical health [40].

Since the adoption of the SDGs in 2015, there has been a more explicit need to link Urban Public Health with the debate around the ecological crisis and sustainable development. Increased attention is being paid to the potential positive synergies between climate mitigation and adaptation measures and health resources [27,41,42,43,44,45]. Reduction of automotive traffic, initiatives for more bicycle- and pedestrian-friendly urban and transportation planning, and the importance of green and blue infrastructure for the improvement of air quality are further examples of such synergies [46,47,48,49,50,51,52]. Possible interventions for more urban green space, for example, are evaluated in terms of co-benefits for climate resilience and human health [49]. Specifically in deprived neighborhoods, interventions should enable access to affordable quality housing, various local facilities, quality open spaces and various mobility options [53]. The Corona pandemic has increased awareness of the importance of quality housing, public spaces, and urban greenery. Questions about the post-Corona city should be linked to questions about health promoting, sustainable, and climate-resilient urban transition [54,55] and reduction of inequalities.

#### 3.1.2. Housing Conditions

Since the pioneering work of social medicine in the 19th century, the spread of communicable diseases has been greatly curbed in industrialized countries [56,57,58]. The catastrophic living and sanitary conditions at the beginning of industrialization [59] have been largely remedied. Meanwhile, following classical problems of hygiene and changing living conditions, other factors have come to the foreground. It is now evident, that physical factors such as temperature, air humidity and ventilation, and building materials contaminated with pollutants have an impact on respiratory health [60,61]. Parasites, fungi, and other pollutants have been known to cause asthma and allergies since the 1980s [62,63,64,65]. At the same time, indirect, intangible factors of housing also have an impact on health [66,67]: the home is perceived as a place of refuge, security, and privacy. It is a constant, a space of daily routines and control over one’s own life, an identity-forming place, possibly tied to social norms and status symbols [66]. The consequences of losing these factors have an impact on health, especially in extreme cases of homelessness [67]. Beyond the evidence that housing conditions are associated with various diseases, these are not simple cause-and-effect relationships, but a complex network of effects [68]. These effects not only translate into increased costs for the health care system, but may also create additional costs in education, crime management, or energy supply [68].

Thus, socioeconomic aspects and the social production of health inequalities are increasingly research issues [69] (pp. 360–361). Almost twenty years after the Ottawa Charter, Mary Shaw still states that despite the strong historical links between housing conditions and health, too much attention is still paid to factors of individual behavior rather than environmental and socioeconomic structures, and that increasing income inequality is inseparable from the problem of lack of affordable housing [67] (p. 414). Numerous factors of poor housing conditions are beyond the direct influence of those who are affected, so an effective solution to these problems must be located at a structural level.

Given the recognition that sociodemographic and socioeconomic conditions may have a stronger influence on poor housing than has long been assumed, there is a need for a fundamental revision of previous (primarily biomedical) established research approaches and methods that justify the link between health, urban planning, social and environmental policy [70]. This would also contribute to more optimal use of available resources [60].

#### 3.1.3. Mobility and Transport Infrastructures

Urban mobility, connectivity and infrastructure are particularly intertwined with urban development and planning. The orientation of urban planning towards the car-friendly city since the 1960s has meanwhile revealed some downsides from the Urban Public Health perspective. The strong research focus since the 1990s on air pollution, (allergic) respiratory diseases, cancer risks, traffic accidents, and possible interventions are expressions of an emerging critique of urban car traffic [71,72,73,74,75,76,77]. The use of research findings from other disciplines is being embraced by Urban Public Health; in particular, the use of transportation and planning research to examine the impact of land use and design on public health [78]. This is due to the recognition that urban and traffic planning of the past decades has “engineered physical activity out of our daily lives.” [78] (p. 89) Urban Public Health perspectives increasingly advocate for urban planning that considers and promotes improving air quality, solving traffic congestion, and increasing overall quality of life in an integrated manner: “Health researchers need to become more involved in environmental research and policy studies, discussion, and decisions about environmental factors […]” [78] (p. 89).

In addition to reduced physical activity, high traffic volume and speeds reduce social contact and contact to goods and services [79]. Transportation infrastructure can connect or disconnect society and thus have impact on social integration, cohesion, and public health [79]. Street connectivity, mixed land use, access to public transportation, pedestrian and bicycle infrastructure, short distances, and traffic safety have been recommended to promote public health and are recognized as effective strategies in creating healthy and sustainable compact cities [35,80,81,82,83,84].

#### 3.1.4. Digitalization

In addition to these facets of infrastructure issues, the most recent aspect is that of digitalization and its potentials and risks for public health and sustainable cities. A lot of potential is seen with regard to health care and the field of eHealth, e.g., smart hospital or an electronic patient record [85]. Digitalization can support the transition from cure to prevention, patients’ empowerment, or healthcare efficiency [86]. The smart city research offers possible synergies with Urban Public Health: sharing economy, electrification and automation, digitalization of different infrastructures can create co-benefits for public health in the form of reduced CO_2_ emissions, new uses for freed-up space, increased traffic space [87,88]. However, technical or even economic barriers to access must be considered as potential disadvantages for more health equity [89]. The question arises about the effects associated with digitalization processes in regard to urban spaces and possible rebound or even negative effects, which must be taken into account. In particular, answering questions about equity, access, (resource-related) sustainability, and the benefits for society will fail without an interdisciplinary approach. Urban Public Health is therefore confronted with the large topic of digitalization as science as a whole. As a phenomenon of societal scale, digitalization is one of the megatrends of the 21st century and potentially generates a great need for research in almost all settings, urban spaces and areas of life.

#### 3.1.5. Climate Change

The discourse around sustainability and the increasing pressure on cities to act, both as a main driver of climate change and as the main addressees for implementing counter-measures, are closely related to the health of the population. Starting with the first United Nations (UN) Climate Change Conference in Rio de Janeiro back in 1992, following the adoption of the UN Sustainable Development Goals (SDGs) in 2015, and the Paris Agreement of the Parties to the Framework Convention on Climate Change (UNFCCC)—cities have become major players in a large-scale socio-ecological transformation.

In the 1990s and early 2000s considerable evidence was generated on the urban environment and the negative health effects of air, water, and soil pollution, noise, exposure to bacteria, viruses, pesticides, and toxins [90,91,92]. Today, this research is integrated into the broader debate on climate change and ecological crisis, that is largely framed around the issues of mobility, resource, and energy transition [49,93]. Climate change favors the mitigation of invasive and potentially health-threatening species, such as Ambrosia in Central Europe or other potentially allergenic plants. Additionally, the predicted increase in vector-based diseases and zoonosis (malaria, dengue fever, rabies, coronaviruses, etc.) are threats to human health [94]. Both increasing urbanization and climate change will further intensify these risks [95]. The appropriate response to these problems raises questions regarding effective interventions: integrated vector management that likewise promotes environmental management, education and awareness, and inter-sectoral collaboration is considered effective and sustainable [96,97,98]. The systematic and regular monitoring of interventions, strategies with more political commitment and social mobilization, exchange of experience and data, pooling of resources, and cooperation would be crucial approaches [99]. Overall, research addressing the health-promoting components of (urban) nature increased, especially in the context of climate mitigation and adaptation measures.

#### 3.1.6. Urban Nature and Ecosystems

Green and blue infrastructures and nature-based solutions offer great potential to be beneficial in three ways: in terms of ecological sustainability, as a health resource, and for greater health equity [49,100]. The health promoting effects of nature and ecosystem services are broadly positioned, e.g., stress-reducing effects, increased physical activity, reducing effects on cardiovascular diseases, and improved mental health [101,102,103,104]. The so-called view that greenery has a relaxing and stress-reducing effect [105,106,107]. Further potential ecosystem services are: food; air quality regulation; climate regulation; water treatment; moderation of disturbance events; erosion prevention; maintenance of soil fertility; maintenance of life cycles and genetic diversity; but also inspiration for culture, art and design; information for cognitive development [103]. Availability, accessibility, but also aesthetics are relevant factors for the active use of urban green areas [108,109]. These findings are worth paying much more attention to the synergies and co-benefits of climate research and health research. Future intervention efforts should focus on these benefits. Or in other words: “It seems reasonable to invest in urban natural environments as a general public health intervention” [101] (p. 381). Urban Public Health could make an important research contribution here, e.g., on questions of the specific design of green spaces, taking into account aspects of access, safety, and quality; or also on questions of the relationship between the degree of biodiversity and human health. In addition, ecological inequalities and environmental justice have received insufficient attention in green space management and urban planning and there is minor attention regarding the links between availability, accessibility, and quality of urban green and socioeconomic inequalities [110] as well as green gentrification.

### 3.2. Social Environment

#### 3.2.1. Overarching Issues

The question of the role of social factors in public health is not new and the associations between poverty, inequality, and lack of education and health are supported with rich evidence [111]. In this paper, these aspects are always considered as crosscutting issues in the social, built and natural environments. The genuinely social-spatial perspective of Urban Public Health, which is inextricably linked to social structures, can broaden the field of research on the social determinants of health. In this sense, Urban Public Health asks about the social structures in a city that shapes the lives of the population, combined with physical structures and their evolution through urbanization itself. In this respects, the socio-spatial and physical structures, as well as urban transformation itself, are investigated as linked to each other. Factors such as demography and inequalities are of particular relevance here.

Apparently, different age groups have partly different demands and needs on their physical environments, e.g., on housing conditions, mobility, or access to public space [112]. Further studies are needed to achieve evidence-based health promotion recommendations that address these needs while tackling inequities [113,114,115,116,117,118].

Tailoring health programs to meet specific needs of population groups (e.g., ethnic, age, gender, minorities) is a recognized key principle of health promotion [119]. Nevertheless, it remains a challenge to ensure barrier- and discrimination-free access to health resources for all population groups [119]. Analyzing health disparities along different indicators is thus an important research focus. In that sense, classical demographic indicators, as well as socioeconomic and socio-cultural indicators and their interconnections with structural factors and the physical environment can help to deconstruct identified disparities and inequalities in urban spaces [120].

#### 3.2.2. Segregation and Gentrification

Socioeconomic disparities and sociocultural differences can be translated in spatial differences [57,58,121]. The connection of social, built, and natural environments is well visible when looking at processes of segregation and gentrification. In the United States, there exists a long-standing research tradition that focuses on aspects of segregation, persistent disadvantage of low-income minority neighborhoods, and racism [122,123]. Racism and discrimination and their manifestation in social structures, condition a range of health consequences and inequalities on at least three levels: “institutionalized policies and practices that maintain racial disadvantage, individual racial discrimination and biased treatment, and internalized cognitive processes” [124] (p. 1140). Systematic housing discrimination and racialized policies that inhibit homeownership for certain population groups have left many neighborhoods in U.S. cities isolated and revealed a geographic pattern of residential segregation [125]. In order to mitigate social and economic adversity, alternative networks or informal structures are often formed in affected neighborhoods to secure the material resources for these disadvantaged population groups. Although segregated, these neighborhoods can show strong internal integration since the homogenous milieu offers social embedding [126]. The centrality of urban land use policies and urban planning for urban public health is visible in such segregation processes and effects [34]. Political decisions about urban planning and development can counteract such processes, or it can stimulate them even further.

Programs and interventions that attempt to break up such structures try to create mixed-income communities, and revitalize disadvantaged urban areas through targeted reinvestment. However, these interventions are often accompanied by adverse effects such as the displacement of low-income urban residents who can no longer afford the rent in revitalized neighborhoods [127]. In such cases, the health of the domestic population will not improve; in fact, it causes stress and illness due to gentrification effects. As a result, the problem is not solved but rather shifted to other neighborhoods. The health equity perspective is often neglected in urban and housing policies, and the importance of the structural context that had led to segregated neighborhoods is often obscured in public discourse [127]. Tulier et al. [122] explored this problem and identified four relevant aspects: (1) neighborhood attributes (infrastructure, economic opportunities/development, social cohesion); (2) individual mechanisms of change (individual health protective resources within a neighborhood experiencing gentrification); (3) neighborhood and individual level mechanisms (economic opportunities and growth, financial status); (4) the role of political and economic institutions (shaping the relationship between gentrification and health).

Gentrification and urban or regional transitions require a deeper understanding of complex macrosocial phenomena and their influence on public health. Studies from the U.S. show that gentrification and displacement are among the most important neighborhood challenges and most common structural psychosocial stressors [125]. Moreover, gentrification and displacement often reinforce and perpetuate existing power structures and asymmetries [123]. For this reason, more attention is necessary on the mediating factors of neighborhood change and health, both those that hinder and those that promote health equity.

#### 3.2.3. Social Cohesion and Networks

Community characteristics of neighborhoods, their importance for physical and mental health of the inhabitants, and the creation of mixed communities are among the approaches to health-promoting urban development [128,129,130,131]. In neighborhoods with weak social cohesion, high levels of violence, and lack of safety, residents are more likely to experience health risks such as sleep deprivation, depression, lack of physical activity, or use of addictive substances [132,133,134,135,136]. Social cohesion thus represents a relevant attribute for health-promoting neighborhoods [135]. Approaches to strengthening social networks and social connectivity is thus increasingly attracting the attention of Urban Public Health [134]. Social cohesion in neighborhoods is closely linked to the built environment and to issues such as mobility and infrastructure as connecting or dividing elements (e.g., intimidating spaces: poorly planned and abandoned places, underpasses, heavily travelled roads). However, associations between social cohesion, health, and urban environments deserve more interdisciplinary research attention [136].

#### 3.2.4. Economic Opportunities and Working Conditions

The urban form, as well as urbanization and urban transformation are largely driven and influenced by economic structures. Urban Public Health has so far paid little attention to these structures, although economic deprivation is recognized as a key driver for health inequalities. The strong correlation between income and health status is transmitted through employment status and contextualized by factors such as gender identity, ethnicity, immigration status, and social class [137,138]. Employment can provide financial security, strengthened social relationships, and increased social status, while precarious employment can also negatively affect all these factors [137,138].

Martins [139] sees the issue of work and employment as relevant to the development of healthy cities in three ways: (i) *Urban Economies*, (ii) *Place(s) of Work*, and (iii) *Work/Economy on Place*. (i) Urban Economies describe the respective degree of diversity of the economic urban system and the mix of production activities of the existing economic sectors, and the extent to which this results in employment opportunities. For example, topics such as local economic development and alternative economies, how it is discussed from the scientific community on sustainable transformation. In addition, processes of structural change in coal regions has implications for health, as well as the future of work in the face of advancing digitalization. (ii) Place(s) of Work describe the analyses of location and spatial distribution of work, related to work routes and movement spaces, and the quality of workspace. This includes new forms of workspaces or alternative/multiple use possibilities, e.g., due digitalization processes. This is followed by the dimension of (iii) Work/Economy in Place, which deals with the shaping of cities or neighborhoods by economy. Retail, which established itself in the city center and thereby promotes social activities and vivid urban life, is related to different consequences when these structures disappear.

Although there is ample evidence of the health effects of economic factors, including employment, an interdisciplinary research approach is needed to generate more knowledge on the links between the economy and health-promoting urban development. In particular, the multi-layered relations between space, employment, urban economy, and health are not limited to the local level of a city or a neighborhood, but are rather integrated in different spatial scales. Furthermore, integrated approaches need to study different economic sectors, and their structures of production and consumption, taking into account aspects of availability and access [140,141].

### 3.3. Governance and Urban Development

Urban governance and development policies shape urban environments and thus effect health. The importance of health policies action that improves urban public health, particularly aiming to reduce inequalities, is emphasized [142]. Data-based information assessed, e.g., by monitoring, surveillance, or health impact assessments are basic tools for an evidence based policy [10]. Analyses of the WHO’s *Healthy Cities Network* shows that cooperation between cities as well as between the various sectors within the city is a key element to tackle inequalities and promote good governance and leadership for health and wellbeing [143,144,145,146]. The benefits and positive contribution of such cooperation networks through mutual knowledge exchange and testing of municipal strategies and interventions have become visible [147,148]. The network has also brought much greater focus to the close linkages between urban development and health, effectively contributing to the dissemination of good practice [149].

Nevertheless, there remains an implementation gap between internationally formulated goals and the actual transformation of our cities. Despite positive developments, this implementation gap, and the successful setting of a strategic and holistic approach in the sense of *Health in All Policies* remains a demanding field of health research. Internal institutional barriers, competing interests, hegemonic values, norms, and processing practices block the path to the policy agenda [149,150,151,152]. Against this background, Urban Public Health has increasingly turned to questions of governance and participation to elevate the potential of broadly involving relevant stakeholders and strengthening participatory processes as effective levers for transforming urban structures and spaces. While citizen participation in designing and implementing health resources, considered a recognized feature of best practice, rarely extends beyond the planning stage [153]. Better understanding of existing governance structures requires more analyses “of the historical, social, and economic processes that have characterized social relations and citizenship in specific local, national, and global contexts” [151] (p. 897), to make the production and reproduction of (power) structures recognizable [151].

## 4. Discussion

The aim of this paper was to compile current evidence regarding the spatial component of health and disease in urban structures and to identify research issues, which are addressing Urban Public Health. Our approach was expanded through a structural lens to the living conditions in urban environments beyond individual health behaviors. Based on an ex-ante developed conceptual approach, we defined selected categories of Urban Public Health to be used for synthesis of the literature. According to these categories, our review emphasizes both the positive and negative impacts of urban structures on health and linkages between urban structures.

With regard to the selected research issues and urban environments, a wide range of further research needs become visible:

- A need for more research on the political structures that impact public health, urban spaces, and the underlying (power) structures.
- With respect to the built and natural environment, there is a need for epidemiological and public health research to link dimensions of the social environment with different spatial scales.
- The housing issue is still predominantly focused on individual behavior instead of exploring socioeconomic structures. Especially the growing pressure on the housing market, cannot be handled by individuals, but must be answered structurally.
- Digitalization processes and concepts such as smart cities need to be critically questioned and studied for their potential as health resources.
- More evidence is required regarding the needs for, quality of, and access to urban nature for all population groups.
- Place-based interventions, which promote and maintain health need to be developed, monitored and evaluated to obtain evidence on health impacts on different population groups in a city.
- Aspects of segregation and gentrification as well as the role of social networks and social cohesion require further evidence on health impacts. Special attention is necessary on the mediating factors of neighborhood change and health.
- It is crucial to take into account both negative and positive factors for health promotion and equity.
- The identification and assessment of dynamic relationships and complex causal processes that shape urban environments [125].
- Urban production and consumption structures, transformation processes of economic structures, economic opportunities in cities and neighborhoods, and their implications for health is a further identified research strand for Urban Public Health.

From a more overarching point of view and the perspective of health promotion, it is essential to include interventions that change urban structures, complementary to the ones that change individual behavior. In addition to analyzing risk factors, it is necessary to analyze urban structures in order to identify deep-seated causes of health and disease [154]. This includes the question of whether and to what extent certain urban systems are health maintaining and/or promoting in their current constitution or what is necessary to bring forth health-promoting potential. Moreover, since urban development is impacted at different scales—from local to global—further phenomena such as globalized markets and resource flows, digitalization and mechanization, migration movements, and climate change need to be examined in terms of Urban Public Health. Especially major issues of this century—urbanization, climate change, and digitalization—have so far been considered from a health perspective only to a limited extent. In particular, the climate crisis is addressed by public health, mainly in terms of risks caused by extreme weather events or invasive species causing (new) infectious diseases. It is essential, however, that Urban Public Health plays a stronger role in shaping climate change mitigation and adaptation measures. Its expertise can and must contribute to urban transformation pathways in terms of social, health, and environmental sustainability. In particular, it must bring in the perspective that urban structures serve also as health resources. In this sense, Urban Public Health has to deal with the challenges of urbanization and the complexity of urban structures.

Another challenge is the categorization of the different environments, as carried out by existing conceptual approaches. Categorization is helpful for systematization and greater clarity. However, it can lead to a pillarization of research with partly disciplinary hegemonies, although there are examples that demonstrated the connections and constitutive relationship between urban environments. A systemic approach is necessary to dissolve this pillar structure, address the complexity of urban structures and to advance public health. We argue that Urban Public Health should take this systemic approach and broaden the existing approaches of public health. Because although public health is already an interdisciplinary field of research, it lacks a broader view with regard to the city and urban environments that the spatial perspective can provide. Existing evidence has provided insights into the different dimensions of health and disease and their distribution in different settings. Now, the task of Urban Public Health is to increasingly contextualize and link these findings. Linking findings, also from different disciplines, and developing spatial thinking can overcome existing single evidence and make other correlations visible, which can then also enable new approaches for interventions. Based on this, Urban Public Health should intensify its research regarding the identification of the causes of health and disease through production and appropriation of space, resulting health outcomes, and their distribution. This research perspective could make a helpful contribution and address Urban Public Health understandings that are still missing or only partly explored. This includes, first, the approach of making health resources of urban structures an explicit research issue, in addition to health risks. Second, it includes research approaches that address urban living conditions and contexts, paying particular attention to spatial relationships, rather than individual health behavior. Third, and here we come more to a conceptual understanding of Urban Public Health, there is a need to develop conceptual approaches to link the public health perspective with spatial perspectives from other scientific disciplines.

## 5. Conclusions

Health takes place within and between urban structures. This makes Urban Public Health a complex and hard-to-grasp field of research. Research to date has already brought much to light in the issues of health risks, but still shows potential in exploring the issues of health resources. This requires interdisciplinary cooperation between public health and various other disciplines, and the development of a common spatial perspective in order to be able to specifically analyze spatial components of health and disease in urban structures. A systemic approach is necessary to develop an understanding of urban development challenges and address complex urban structures that influence health. Urban Public Health, as an interdisciplinary field, can enable different disciplines to incorporate in their approaches an understanding of public health and especially its broadened understanding of health as resource.

This is a perspective that should also be increasingly taken into account in (urban) politics and policy making. A modified understanding of health and the idea of health resources can be made fruitful for cities and urban development. In particular, concepts such as sustainability strategies, climate protection and climate adaptation plans should integrate such a health perspective across all fields of action—in line with the WHO’s Health in All Policies approach.

Focus on health resources and the transformation of urban structures rather than behavior, opens up remarkable potential for an overall societal change. Urban Public Health should contribute to urban environments, which maintain and promote health and make the city a healthy, just, and sustainable place. A perspective of Urban Public Health, as presented in this paper, means to give the inhabitants of a city the opportunity to shape their living environment in a self-determined and healthy way. Moreover, it would enable structural alternatives to the dominant pathogenic understanding of health and the health care system. This implies nothing less than raising and advancing the emancipatory potential for free and equal urban inhabitants [20].

## Figures and Tables

**Table 1:** Selected categories of Urban Public Health.

Built and Natural Environment	Social Environment
Housing conditions	Segregation and gentrification
Mobility and transport infrastructures	Social cohesion and networks
Digitalization	Economic opportunities and working conditions
Climate change	
Urban nature and ecosystems	
**Governance and Urban Development**	

## Data Availability

Not applicable.

## Appendix A

**Table A1:** Main Literature for analysis.

Author	Year	Title	Type of Research	Objectives	Categories of Urban Public Health
WHO Regional Office for Europe.	2010	Urban planning, environment and health: From evidence to policy action.	Report	(a) Summary notes of an expert meeting held in fall 2008, (b) evidence review on the urban planning impacts on environment and health, and (c) summary reports of two case study cities on their local priority challenges.	Built and Natural Environment - Overarching issues -
Glasgow Centre for Population Health	2013	The built environment and health: an evidence review.	Review	To summarize the main ways in which built environment features and neighborhood characteristics can impact on health and wellbeing.	Built and Natural Environment - Overarching issues -
Renalds, A.; Smith, T.H.	2010	A systematic review of built environment and health.	Systematic Review	To compile the published research that examined the relationship between built environment and health.	Built and Natural Environment - Overarching issues -
Lee, Y.S.	1994	Urban planning and vector control in Southeast Asian cities.	Review	To rethink intersectoral and integrated approaches to the design and planning of healthy urban environments, covering such matters as basic infrastructure and services, urban land use planning and waste management, health promoting housing and architecture, and the control of all other factors that determine human health and wellbeing.	Built and Natural Environment - Overarching issues -
World Health Organization	2011	Global Status Report on Noncommunicable Diseases 2010	Report	Description of the global burden of NCDs, their risk factors and determinants.	Built and Natural Environment - Overarching issues -
World Health Organization	2020	Noncommunicable Diseases: Progress Monitor 2020	Report	Progress monitoring.	Built and Natural Environment - Overarching issues -
Berry, H.L.	2007	‘Crowded suburbs’ and ‘killer cities’: a brief review of the relationship between urban environments and mental health.	Review	To review recent studies linking disadvantaged urban environments with mental health.	Built and Natural Environment - Overarching issues -
Schilling, J.; Linton, L.S.	2005	The public health roots of zoning: in search of active living’s legal genealogy.	Research Article	To understand the dynamic nature of land use law and policy, and how policymakers might accomplish zoning reform to encourage more physically active environments, this paper traces the public health roots of zoning through a family tree of land-use legal doctrines.	Built and Natural Environment - Overarching issues - Social Environment - Segregation and Gentrification -
Gomez, L.F.; Sarmiento, R.; Ordoñez, M.F.; Pardo, C.F.; Sá, T.H. de; Mallarino, C.H.; Miranda, J.J.; Mosquera, J.; Parra, D.C.; Reis, R.; et al.	2015	Urban environment interventions linked to the promotion of physical activity: a mixed methods study applied to the urban context of Latin America.	Mixed Methods Review	Summarizes the evidence from quantitative systematic reviews that assessed the association between urban environment attributes and physical activity. It also documents sociopolitical barriers and facilitators involved in urban interventions linked with active living.	Built and Natural Environment - Overarching issues - Mobility and Transport Infrastructures
United Nations	2019	World Population Prospects 2019: Highlights; Statistical Papers.	Report	Presents population estimates from 1950 to the present for 235 countries or areas, underpinned by analyses of historical demographic trends.	Built and Natural Environment - Overarching issues -
Gelormino, E.; Melis, G.; Marietta, C.; Costa, G.	2015	From built environment to health inequalities: An explanatory framework based on evidence.	Scoping Review	Carry out evidence on the built environment and its health equity impact.	Built and Natural Environment - Overarching issues -
Dendup, T.; Feng, X.; Clingan, S.; Astell-Burt, T.	2018	Environmental Risk Factors for Developing Type 2 Diabetes Mellitus: A Systematic Review.	Systematic Review	Evidence on the environmental determinants of T2DM	Built and Natural Environment - Overarching issues -
Kim, D.H.; Yoo, S.	2019	How Does the Built Environment in Compact Metropolitan Cities Affect Health? A Systematic Review of Korean Studies.	Systematic Review	Associations between health-related outcomes and the built environment characteristics of compact metropolitan cities.	Built and Natural Environment - Overarching issues -
Hassan, N.A.; Hashim, Z.; Hashim, J.H.	2016	Impact of Climate Change on Air Quality and Public Health in Urban Areas.	Review	How climates undergo changes and the effect of climate change on air quality as well as public health.	Built and Natural Environment - Overarching issues -
Slovic, A.D.; de Oliveira, M.A.; Biehl, J.; Ribeiro, H.	2016	How Can Urban Policies Improve Air Quality and Help Mitigate Global Climate Change: a Systematic Mapping Review.	Systematic Mapping Review	Overview of local air pollution control policies and programs that aim to reduce air pollution levels in megacities, and evidence measuring their efficacy.	Built and Natural Environment - Overarching issues -
Bartholy, J.; Pongrácz, R.	2018	A brief review of health-related issues occurring in urban areas related to global warming of 1.5 °C.	Review	To summarize and provide an overview of a representative selection of recent studies that specifically focus on the actual health issues in urban areas and also address global warming related consequences at the same time.	Built and Natural Environment - Overarching issues -
Ali, M.U.; Liu, G.; Yousaf, B.; Ullah, H.; Abbas, Q.; Munir, M.A.M.	2019	A systematic review on global pollution status of particulate matter-associated potential toxic elements and health perspectives in urban environment.	Systematic Review	(1) The possible natural and anthropogenic emission sources of PM and associated PTEs, (2) PM resuspension mechanism and particle size behavior in atmosphere, (3) pollution status of particulate matter and PTEs in different countries worldwide, (4) human exposure and health risk associated with the increased pollution level of PM and associated PTEs.	Built and Natural Environment - Overarching issues -
Kruize, H.; van der Vliet, N.; Staatsen, B.; Bell, R.; Chiabai, A.; Muiños, G.; Higgins, S.; Quiroga, S.; Martinez-Juarez, P.; Aberg Yngwe, M.; et al.	2019	Urban Green Space: Creating a Triple Win for Environmental Sustainability, Health, and Health Equity through Behavior Change.	Narrative Literature Review	To explore potential wins of urban green space for environmental sustainability, health, and health equity and to investigate how to increase the use of urban green space through behavior change.	Built and Natural Environment - Overarching issues - - Climate Change - - Urban Nature and Ecosystems -
Felappi, J.F.; Sommer, J.H.; Falkenberg, T.; Terlau, W.; Kötter, T.	2020	Green infrastructure through the lens of “One Health”: A systematic review and integrative framework uncovering synergies and trade-offs between mental health and wildlife support in cities.	Systematic Review	To compile urban green space’s characteristics that affect mental health and urban wildlife support, and then identify potential synergies and trade-offs between these dimensions.	Built and Natural Environment - Overarching issues -
Nieuwenhuijsen, M.J.	2020	Urban and transport planning pathways to carbon neutral, liveable, and healthy cities: A review of the current evidence.	Narrative Meta-Review	How to improve public health through better urban and transport planning.	Built and Natural Environment - Overarching issues -
Trojanowska, M.	2021	Urban design and therapeutic landscapes. Evolving theme.	Literature Review, Field Observation, Conceptual Framework	Attempts to create health-promoting places. The question is what are the architectural features linked to health promotion?	Built and Natural Environment - Overarching issues -
Romanello, M.; McGushin, A.; Di Napoli, C.; Drummond, P.; Hughes, N.; Jamart, L.; Kennard, H.; Lampard, P.; Solano Rodriguez, B.; Arnell, N.; et al.	2021	The 2021 report of the Lancet Countdown on health and climate change: code red for a healthy future.	Review	To monitor the health impacts of climate change, and the progress, or absence, in the world’s response.	Built and Natural Environment - Overarching issues -
Sverdlik, A.	2011	Ill-health and poverty: a literature review on health in informal settlements.	Literature Review	Health in informal settlements that now house a substantial proportion of the urban population in Africa, Asia, and Latin America.	Built and Natural Environment - Housing Conditions - Social Environment - Segregation and Gentrification -
Ezeh, A.; Oyebode, O.; Satterthwaite, D.; Chen, Y.-F.; Ndugwa, R.; Sartori, J.; Mberu, B.; Melendez-Torres, G.J.; Haregu, T.; Watson, S.I.; et al.	2017	The history, geography, and sociology of slums and the health problems of people who live in slums.	Systematic Review	(1) Provide some background to slums covering terminology and definitions, the size of slum populations, and the dynamics of their growth. (2) A theoretical argument that slum health should be a substantive topic for study, distinct from urban health, and from poverty and health. (3) To examine the extent and nature of previous research in slum health. (4) To describe the physical and social factors affecting health in slums. (5) Describe the particular health problems of people who live in slums.	Built and Natural Environment - Housing Conditions - Social Environment - Segregation and Gentrification -
Matte, T.D.; Jacobs, D.E.	2000	Housing and health-current issues and implications for research and programs.	Review	Overview of the ways in which the home environment can affect human health, and to describe how specific health hazards in housing are related, and considers implications of these concerns for research and programs to address the health-housing connection.	Built and Natural Environment - Housing Conditions -
Krieger, J.; Higgins, D.L.	2002	Housing and Health: Time Again for Public Health Action.	Review	To describe some of the evidence linking housing conditions to health, place public health’s role in addressing housing issues in an historical context, provide examples of contemporary local public health activities in the housing arena, and conclude with suggestions for public health action in the next decade.	Built and Natural Environment - Housing Conditions -
Malveaux, F.J.; Fletcher-Vincent, S.A.	1995	Environmental risk factors of childhood asthma in urban centers.	Review	Asthma morbidity and mortality of children in urban areas.	Built and Natural Environment - Housing Conditions -
Grant, E.N.; Alp, H.; Weiss, K.B.	1999	The challenge of inner-city asthma.	Review	Key risk factors contributing to asthma morbidity within the inner city and opportunities for successful intervention strategies.	Built and Natural Environment - Housing Conditions -
LeNoir, M.A.	1999	Asthma in Inner Cities.	Review	To discuss the problem of asthma in inner cities regarding barriers to optimal management of patients with asthma and why certain populations in the United States suffer and die more often.	Built and Natural Environment - Housing Conditions -
Tartasky, D.	1999	Asthma in the inner city: a growing public health problem.	Review	The epidemiology of asthma in urban areas and various risk factors that are important in achieving control of this disease. Suggestions for future interventions are discussed.	Built and Natural Environment - Housing Conditions -
Evans, G.W.	2003	The built environment and mental health.	Review	Critically analyzes what is known about the built environment and mental health.	Built and Natural Environment - Housing Conditions -
Shaw, M.	2004	Housing and public health.	Review	Considers the broad area of housing and public health, taking into account the range of factors through which housing affects health.	Built and Natural Environment - Housing Conditions -
Ambrose, P.J.	2001	Living conditions and health promotion strategies.	Review	Assesses the empirical evidence concerning the interface between living conditions and health status provided by a number of case studies of urban regeneration in London, and Brighton and Hove.	Built and Natural Environment - Housing Conditions -
Dunn, J.R.	2000	Housing and Health Inequalities: Review and Prospects for Research.	Review	To extend possibilities for housing and health research by drawing connections between, and identifying limitations of, well-researched dimensions of housing on the one hand, and research emphases within population health on the other. In so doing, the paper identifies new points of emphasis for future housing and health research.	Built and Natural Environment - Housing Conditions -
Hemminki, K.; Pershagen, G.	1994	Cancer risk of air pollution: epidemiological evidence.	Review	Epidemiological evidence on air pollution-induced and engine exhaust-induced cancer.	Built and Natural Environment - Mobility and Transport Infrastructures -
D’Amato, G.	1999	Outdoor air pollution in urban areas and allergic respiratory diseases.	Review	Respiratory allergic diseases in urban and industrialized areas.	Built and Natural Environment - Mobility and Transport Infrastructures -
Bunn, F.; Collier, T.; Frost, C.; Ker, K.; Roberts, I.; Wentz, R.	2003	Area-wide traffic calming for preventing traffic related injuries.	Systematic Review	To assess the effects of area-wide traffic calming for preventing traffic related crashes, injuries, and deaths.	Built and Natural Environment - Mobility and Transport Infrastructures -
Egan, M.; Petticrew, M.; Ogilvie, D.; Hamilton, V.	2003	New roads and human health: a systematic review.	Systematic Review	To synthesize evidence of the health effects of construction of new roads by systematically reviewing observational studies of such effects.	Built and Natural Environment - Mobility and Transport Infrastructures -
Saelens, B.E.; Sallis, J.F.; Frank, L.D.	2003	Environmental correlates of walking and cycling: findings from the transportation, urban design, and planning literatures.	Review	Neighborhood environment characteristics proposed to be relevant to walking/cycling for transport are defined, including population density, connectivity, and land use mix.	Built and Natural Environment - Mobility and Transport Infrastructures -
Mindell, J.S.; Karlsen, S.	2012	Community severance and health: what do we actually know?	Systematic Review	To discusses the published evidence relating to community severance, the extent of evidence on its effects on health, and how severance might be quantified.	Built and Natural Environment - Mobility and Transport Infrastructures -
Blečić, I.; Congiu, T.; Fancello, G.; Trunfio, G.A.	2020	Planning and Design Support Tools for Walkability: A Guide for Urban Analysts.	Review	Survey of operational methods for walkability analysis and evaluation, which we hold show promise as decision-support tools for sustainability-oriented planning and urban design.	Built and Natural Environment - Mobility and Transport Infrastructures -
Devarajan, R.; Prabhakaran, D.; Goenka, S.	2020	Built environment for physical activity-An urban barometer, surveillance, and monitoring.	Review	It provides the developing country climate sensitive multidisciplinary perspective embedded in the existing knowledge of physical activity and built environment. It develops a framework for a dynamic urban barometer with relevant indicators, inclusive of the developing country perspective, which would reflect the progress and status of different countries’, cities’ and towns’ built environment, and the related polices.	Built and Natural Environment - Mobility and Transport Infrastructures -
Stankov, I.; Garcia, L.M.T.; Mascolli, M.A.; Montes, F.; Meisel, J.D.; Gouveia, N.; Sarmiento, O.L.; Rodriguez, D.A.; Hammond, R.A.; Caiaffa, W.T.; et al.	2020	A systematic review of empirical and simulation studies evaluating the health impact of transportation interventions.	Systematic Review	To understand health impacts of transportation initiatives, conducting a systematic review of longitudinal health evaluations involving: (a) bus rapid transit (BRT); (b) bicycle lanes; (c) Open Streets programs; and (d) aerial trams/cable cars.	Built and Natural Environment - Mobility and Transport Infrastructures -
Giles-Corti, B.; Vernez-Moudon, A.; Reis, R.; Turrell, G.; Dannenberg, A.L.; Badland, H.; Foster, S.; Lowe, M.; Sallis, J.F.; Stevenson, M.; et al.	2016	City planning and population health: a global challenge.	Review	Health impacts of city planning through transport mode choices.	Built and Natural Environment - Mobility and Transport Infrastructures -
Uslu, A.M.; Stausberg, J.	2008	Value of the electronic patient record: an analysis of the literature.	Systematic Review	Whether and to what extent the use of an Electronic Patient Record is worthwhile.	Built and Natural Environment - Digitalization -
Odone, A.; Buttigieg, S.; Ricciardi, W.; Azzopardi-Muscat, N.; Staines, A.	2019	Public health digitalization in Europe.	Theory-based Analysis	To reflect on the potential of applying digital tools to public health and discuss some key challenges.	Built and Natural Environment - Digitalization -
Gibbons, M.C.	2005	A historical overview of health disparities and the potential of eHealth solutions.	Historical Overview	Health disparities in the United States and Europe.	Built and Natural Environment - Digitalization -
Buttazzoni, A.; Veenhof, M.; Minaker, L.	2020	Smart City and High-Tech Urban Interventions Targeting Human Health: An Equity-Focused Systematic Review.	Systematic Review	To document and analyze the inclusion of equity considerations and dimensions in smart city interventions aimed to improve human health and wellbeing.	Built and Natural Environment - Digitalization -
Creutzig, F.; Franzen, M.; Moeckel, R.; Heinrichs, D.; Nagel, K.; Nieland, S.; Weisz, H.	2019	Leveraging digitalization for sustainability in urban transport.	Review	Governance of Smart Mobility.	Built and Natural Environment - Digitalization -
Landrigan, P.J.; Claudio, L.; Markowitz, S.B.; Berkowitz, G.S.; Brenner, B.L.; Romero, H.; Wetmur, J.G.; Matte, T.D.; Gore, A.C.; Godbold, J.H.; et al.	1999	Pesticides and inner-city children: exposures, risks, and prevention.	Review	(a) To review data on children’s exposures to pesticides with emphasis on exposures in the inner-city and their relation to issues of environmental justice. (b) To review data on the vulnerability of children to pesticides, with particular reference to the developmental toxicity of chlorpyrifos and of certain pyrethroids; and (c) To consider the current state of neurodevelopmental toxicity testing for pesticides and to review the adequacy and sensitivity of current test procedures.	Built and Natural Environment - Climate Change -
Wong, C.S.C.; Li, X.; Thornton, I.	2006	Urban environmental geochemistry of trace metals.	Review	To provide an overview of the development of urban environmental geochemistry as a field of scientific study and highlight major transitions during the course of its development from its establishment to the major scientific interests in the field.	Built and Natural Environment - Climate Change -
Crane, M.; Lloyd, S.; Haines, A.; Ding, D.; Hutchinson, E.; Belesova, K.; Davies, M.; Osrin, D.; Zimmermann, N.; Capon, A.; et al.	2021	Transforming cities for sustainability: A health perspective.	Review	This paper sets out to determine how change in urban settings could be brought about to achieve health and environmental goals synergistically, taking into account the potential trade-offs of focusing exclusively on either health or environmental issues.	Built and Natural Environment - Climate Change -
Ebi, K.L.; Harris, F.; Sioen, G.B.; Wannous, C.; Anyamba, A.; Bi, P.; Boeckmann, M.; Bowen, K.; Cissé, G.; Dasgupta, P.; et al.	2020	Transdisciplinary Research Priorities for Human and Planetary Health in the Context of the 2030 Agenda for Sustainable Development.	Review	It outlines a research agenda to address cross-cutting knowledge gaps for further understanding and management of the health risks of global environmental changes through an expert consultation and review process.	Built and Natural Environment - Climate Change -
Eder, M.; Cortes, F.; Teixeira de Siqueira Filha, N.; Araújo de França, G.V.; Degroote, S.; Braga, C.; Ridde, V.; Turchi Martelli, C.M.	2018	Scoping review on vector-borne diseases in urban areas: transmission dynamics, vectorial capacity and co-infection.	Scoping Review	To identify knowledge gaps on transmission dynamics, vectorial capacity, and co-infections regarding VBDs in urban areas.	Built and Natural Environment - Climate Change -
Salgado, M.; Madureira, J.; Mendes, A.S.; Torres, A.; Teixeira, J.P.; Oliveira, M.D.	2020	Environmental determinants of population health in urban settings. A systematic review.	Systematic Review	Key environmental determinants and respective dimensions and indicators, relevant to evaluate population health in urban settings, and to understand their potential implications into policies.	Built and Natural Environment - Climate Change -
Degroote, S.; Zinszer, K.; Ridde, V.	2018	Interventions for vector-borne diseases focused on housing and hygiene in urban areas: a scoping review.	Scoping Review	To conduct a review on VBD interventions relevant to housing and hygiene in urban areas.	Built and Natural Environment - Climate Change -
Marcos-Marcos, J.; Olry de Labry-Lima, A.; Toro-Cardenas, S.; Lacasaña, M.; Degroote, S.; Ridde, V.; Bermudez-Tamayo, C.	2018	Impact, economic evaluation, and sustainability of integrated vector management in urban settings to prevent vector-borne diseases: a scoping review.	Scoping Review	To identify components related to impacts, economic evaluation, and sustainability that might contribute to an integrated approach to VBD prevention.	Built and Natural Environment - Climate Change -
Otmani Del Barrio, M.; Simard, F.; Caprara, A.	2018	Supporting and strengthening research on urban health interventions for the prevention and control of vector-borne and other infectious diseases of poverty: scoping reviews and research gap analysis.	Review	To describe the collaboration and partnership of the Special Programme for Research and Training in Tropical Diseases (TDR) hosted by the World Health Organization (WHO) and the “VEctor boRne DiseAses Scoping reviews” (VERDAS) Research Consortium as they joined efforts in response to filling the gap in knowledge and evidence by supporting the development of a series of scoping reviews that highlight priority research gaps and policy implications to address vector-borne and other infectious diseases at the urban level.	Built and Natural Environment - Climate Change -
van den Bosch, M.; Ode Sang, Å.	2017	Urban natural environments as nature-based solutions for improved public health—A systematic review of reviews.	Systematic Review of Reviews	To evaluate the evidence on public health benefits of exposure to natural environments and explore how this knowledge could be framed within the NBS concept.	Built and Natural Environment - Urban Nature and Ecosystems -
Wolf, K.L.; Lam, S.T.; McKeen, J.K.; Richardson, G.R.A.; van den Bosch, M.; Bardekjian, A.C.	2020	Urban Trees and Human Health: A Scoping Review.	Scoping Review	To examine how urban trees affect human health.	Built and Natural Environment - Urban Nature and Ecosystems -
Semeraro, T.; Scarano, A.; Buccolieri, R.; Santino, A.; Aarrevaara, E.	2021	Planning of Urban Green Spaces: An Ecological Perspective on Human Benefits.	Review	To provide an overview of the benefits and limitations of applying an ecosystem services approach in designing GI, focusing on green roofs and community gardens.	Built and Natural Environment - Urban Nature and Ecosystems -
Sallis, J.F.; Floyd, M.F.; Rodríguez, D.A.; Saelens, B.E.	2012	Role of built environments in physical activity, obesity, and cardiovascular disease.	Review	To describe multilevel ecological models of behavior as they apply to physical activity, describe key concepts, summarize evidence on the relation of built environment attributes to physical activity and obesity, and provide recommendations for built environment changes that could increase physical activity.	Built and Natural Environment - Urban Nature and Ecosystems -
Braubach, M.; Egorov, A.; Mudu, P.; Wolf, T.; Ward Thompson, C.; Martuzzi, M.	2017	Effects of Urban Green Space on Environmental Health, Equity and Resilience.	Review	Pathways that link green spaces to health and wellbeing, and discusses available evidence of specific beneficial effects such as improved mental health, reduced risks of cardiovascular disease, obesity, diabetes and death, and improved pregnancy outcomes.	Built and Natural Environment - Urban Nature and Ecosystems -
Schüle, S.A.; Hilz, L.K.; Dreger, S.; Bolte, G.	2019	Social Inequalities in Environmental Resources of Green and Blue Spaces: A Review of Evidence in the WHO European Region.	Systematic Review	To synthesize evidence of environmental inequalities, focusing on availability and accessibility measures of green and blue spaces	Built and Natural Environment - Urban Nature and Ecosystems -
Allender, S.; Foster, C.; Hutchinson, L.; Arambepola, C.	2008	Quantification of urbanization in relation to chronic diseases in developing countries: a systematic review.	Systematic Review	To understand how urbanization has been measured in studies which examined chronic disease as an outcome.	Social Environment - Overarching issues -
Li, F.	2016	Physical activity and health in the presence of China’s economic growth: Meeting the public health challenges of the aging population.	Review	To provide a broad perspective on the impact of rapid economic development, industrialization, and urbanization on health-related behaviors, with a specific focus on physical activity among older adults. Specifically, an overview of the demographic context, significant public health challenges, evidence on physical activity and exercise interventions.	Social Environment - Overarching issues -
Kusuma, Y.S.; Babu, B.V.	2018	Migration and health: A systematic review on health and health care of internal migrants in India.	Systematic Review	To review various health conditions and health care access of internal migrants in India.	Social Environment - Overarching issues -
Robbins, R.N.; Scott, T.; Joska, J.A.; Gouse, H.	2019	Impact of urbanization on cognitive disorders.	Review	To examine cognitive disorders and urbanization.	Social Environment - Overarching issues -
Eckert, S.; Kohler, S.	2014	Urbanization and health in developing countries: a systematic review.	Systematic Review	Urban–rural and intra-urban health differences in developing countries and whether a health advantage can be found for urban areas.	Social Environment - Overarching issues -
Freudenberg, N.	2000	Health promotion in the city: a review of current practice and future prospects in the United States.	Review	Several common strategies for health promotion are described, and their relevance to the unique characteristics of urban populations is assessed.	Social Environment - Overarching issues -
Bhavsar, N.A.; Kumar, M.; Richman, L.	2020	Defining gentrification for epidemiologic research: A systematic review.	Systematic Review	The current state of literature describing the association between gentrification and health.	Social Environment - Segregation and Gentrification -
Tulier, M.E.; Reid, C.; Mujahid, M.S.; Allen, A.M.	2019	“Clear action requires clear thinking”: A systematic review of gentrification and health research in the United States.	Systematic Review	To systematically evaluate how empirical studies have addressed questions regarding the relationship between gentrification and health and wellness conceptually and methodologically.	Social Environment - Segregation and Gentrification -
Smith, G.S.; Breakstone, H.; Dean, L.T.; Thorpe, R.J.	2020	Impacts of Gentrification on Health in the US: a Systematic Review of the Literature.	Systematic Review	To synthesize findings from US population-based, peer-reviewed studies which examine associations between gentrification and health, highlighting both needs and strengths of existing research, and provide suggestions for expanding this body of work.	Social Environment - Segregation and Gentrification -
Fiscella, K.; Williams, D.R.	2004	Health disparities based on socioeconomic inequities: implications for urban health care.	Review	To discuss health disparities based on socioeconomic status in the context of urban health care.	Social Environment - Segregation and Gentrification -
Schnake-Mahl, A.S.; Jahn, J.L.; Subramanian, S.V.; Waters, M.C.; Arcaya, M.	2020	Gentrification, Neighborhood Change, and Population Health: a Systematic Review.	Systematic Review	To better understand of how neighborhood socioeconomic and cultural changes impact equity, specifically disparities in health and health care access.	Social Environment - Segregation and Gentrification -
Diez Roux, A.V.	2003	Residential environments and cardiovascular risk.	Review	Existing empirical research relating residential environments to cardiovascular outcomes and risk factors is summarized.	Social Environment - Social Cohesion and Networks -
Diez Roux, A.V.; Mujahid, M.S.; Hirsch, J.A.; Moore, K.; Moore, L.V.	2016	The Impact of Neighborhoods on CV Risk.	Review	Summarize the approaches used to characterize residential neighborhood environments in the MESA cohort, provides an overview of key findings to date, and discusses challenges and opportunities in neighborhood health effects research.	Social Environment - Social Cohesion and Networks -
Gomez, L.F.; Soto-Salazar, C.; Guerrero, J.; Garcia, M.; Parra, D.C.	2020	Neighborhood environment, self-rated health and quality of life in Latin America.	Systematic Review	The associations between neighborhood environments and self-rated health (SRH) and health-related quality of life (HR-QOL) in the urban context of Latin America.	Social Environment - Social Cohesion and Networks -
Pinto, A.D.; Hassen, N.; Craig-Neil, A.	2018	Employment Interventions in Health Settings: A Systematic Review and Synthesis.	Systematic Review	To identify both studies of employment interventions in health care settings and common characteristics of successful interventions.	Social Environment - Economic Opportunities and Working Conditions -
Hult, M.; Lappalainen, K.; Saaranen, T.K.; Räsänen, K.; Vanroelen, C.; Burdorf, A.	2020	Health-improving interventions for obtaining employment in unemployed job seekers.	Intervention Review	To assess the effectiveness of health-improving interventions for obtaining employment in unemployed job seekers.	Social Environment - Economic Opportunities and Working Conditions -
Hollands, G.J.; Carter, P.; Anwer, S.; King, S.E.; Jebb, S.A.; Ogilvie, D.; Shemilt, I.; Higgins, J.P.T.; Marteau, T.M.	2019	Altering the availability or proximity of food, alcohol, and tobacco products to change their selection and consumption.	Intervention Review	1. To assess the impact on selection and consumption of altering the availability or proximity of: (a) food (including non-alcoholic beverages), (b) alcohol, and (c) tobacco products. 2. To assess the extent to which the impact of these interventions is modified by characteristics of: (i) studies, (ii) interventions, and (iii) participants.	Social Environment - Economic Opportunities and Working Conditions -
Burris, S.; Hancock, T.; Lin, V.; Herzog, A.	2007	Emerging strategies for healthy urban governance.	Thematic Review	To describe the concept of governance, distinguishing between reforms aimed at improving how government works and innovations that more fundamentally reinvent governance by developing new institutions and processes of local stakeholder control.	Governance and Urban Development
Flynn, B.C.	1996	Healthy Cities: toward worldwide health promotion.	Review	To describe the dynamic status of Healthy Cities globally and summarizes what is known about these efforts.	Governance and Urban Development
Quilling, E.; Kruse, S.; Kuchler, M.; Leimann, J.; Walter, U.	2020	Models of Intersectoral Cooperation in Municipal Health Promotion and Prevention: Findings from a Scoping Review.	Scoping Review	This paper deals with models of intersectoral cooperation in municipal health promotion. It frames the methodology and the central results of a literature and database search.	Governance and Urban Development
Green, G.	2013	Age-friendly cities of Europe.	Review	To summarize how members of the European Healthy Cities Network have applied the ‘healthy ageing’ approach developed by the World Health Organization in their influential report on Active Ageing.	Governance and Urban Development
Green, G.	2012	Intersectoral planning for city health development.	Review	The evolution and process of City Health Development Planning (CHDP) in municipalities participating in the European Network of Healthy Cities organized by the European Region of the World Health Organization.	Governance and Urban Development
Barton, H.; Grant, M.	2013	Urban planning for healthy cities. A review of the progress of the European Healthy Cities Programme.	Review	To evaluate the progress made by European cities in relation to Healthy Urban Planning (HUP) during Phase IV of the World Health Organization’s Healthy Cities programme (2003–2008).	Governance and Urban Development
Heritage, Z.; Green, G.	2013	European national healthy city networks: the impact of an elite epistemic community.	Review	Using the concept of epistemic communities, the evolution and impact of NNs is considered, as is their future development.	Governance and Urban Development
Barten, F.; Akerman, M.; Becker, D.; Friel, S.; Hancock, T.; Mwatsama, M.; Rice, M.; Sheuya, S.; Stern, R.	2011	Rights, knowledge, and governance for improved health equity in urban settings.	Review	Focuses on governance to address the social and environmental determinants of urban health inequities. It outlines the key components of governance and the plausible pathways to urban health inequity.	Governance and Urban Development
Korfmacher, K.S.; Aviles, K.; Cummings, B.J.; Daniell, W.; Erdmann, J.; Garrison, V.	2014	Health impact assessment of urban waterway decisions.	Review	Presents four recent HIAs of water-related decisions in the United States and Puerto Rico.	Governance and Urban Development
Katz, A.S.; Cheff, R.M.; O’Campo, P.	2015	Bringing stakeholders together for urban health equity: hallmarks of a compromised process.	Review	Discussion regarding the utility of participation processes in advancing urban health equity by closely reading the processes themselves.	Governance and Urban Development
